# Performance of a Risk Analytic Tool (Index of Tissue Oxygen Delivery “IDO2”) in Pediatric Cardiac Intensive Care Unit of a Developing Country

**DOI:** 10.3389/fped.2022.846074

**Published:** 2022-06-03

**Authors:** Qalab Abbas, Muhammad Zaid H. Hussain, Fatima Farrukh Shahbaz, Naveed ur Rehman Siddiqui, Babar S. Hasan

**Affiliations:** ^1^Department of Pediatrics and Child Health, Aga Khan University, Karachi, Pakistan; ^2^Medical College, Aga Khan University, Karachi, Pakistan

**Keywords:** children, cardiac intensive care unit, developing country, cardiac surgery, risk estimates

## Abstract

**Objective:**

To determine the performance of a commercially available risk analytic tool (IDO2) to estimate the risk for SVO2 < 40% in patients admitted in cardiac intensive care unit (CICU).

**Methods:**

Medical and T3 records of all patients (aged 1 day to 12 years, weight >2 kg) who received care in the CICU between October 1st, 2019 and October 1st, 2020, had SvO2 lab(s) drawn during CICU course and whose data was transmitted to T3, were included. The average IDO2 Index was computed in the 30-min period immediately prior to each SvO2 measurement and used as a predictor score for SvO2 < 40%.

**Results:**

A total of 69 CICU admissions from 65 patients, median age 9.3 months (interquartile range 20.8) were identified. Surgical and medical patients were 61 (88%) and 8 (12%) respectively; 4 (5.7%) patients had single ventricle physiology. Tetralogy of Fallot *n* = 23 (33.3%) and ventricular septal defects 17 (24.6%) were major cardiac diagnosis. Sixty-one (89.9%) of the admissions were successfully discharged from the hospital. Of the 187-total included SvO2 labs, 17 (9%) were <40%. The AUC of estimating SvO2 < 40% IDO2 was 0.87 [confidence interval (CI): 0.79–0.94]. Average IDO2 above 75 had the highest absolute risk (42.11, CI: 20.25–66.50) and highest RR (4.63, CI: 2.31–9.28, *p*-value < 0.0001) of SvO2 < 40%.

**Conclusion:**

IDO2 performed well in estimating low SvO2 (<40%) in pediatric patients presenting to a CICU in a low resource setting. Future work is needed to determine the effect of this risk analytic tool on clinical outcomes in such a setting.

## Introduction

Postoperative care of children undergoing congenital heart disease (CHD) surgery demands constant interpretation of high velocity, high volume data coming from many sources (physiologic, laboratory data etc.) and subsequent decision making based on the interpretation of these inputs. In low middle income countries (LMICs), this is complicated due to a low provider to patient ratio, limited expertise, and the complex clinical states of patients' (i.e., delayed diagnosis or referral, comorbidities like malnutrition and infections etc.) (1–6). All these factors make the postoperative period extremely vulnerable and complex, precluding early identification of inadequate tissue oxygen delivery which can lead to increased morbidity and mortality (2, 7). If this abnormal tissue oxygen delivery can be identified early coupled with a frame work of intervention to address it, the outcomes can be changed for the better (8). The high- frequency, velocity, and volume data originating from patients can be used for real-time analysis and continuous assessment of patients condition and risk estimation. Predictive modeling and continuous estimation of probabilities of a patients' physiologic state can be one way to integrate diverse, physiologic signals, and provide an early warning system related to the trajectory of a patient in response to their clinical state or their response to specific treatments. One such algorithm, recently FDA 510(k) cleared and displayed at the bedside by the T3 (tracking trajectory and trigger) Visualization Platform (Etiometry, Inc., Boston, MA), is the Inadequate oxygen delivery (IDO2) index (4).

T3 is a novel patient data aggregation, visualization, and risk analytic tool. It captures data from patients monitors, ventilators, and the laboratory information system (4). The IDO2 algorithm utilizes up to 10 physiologic values [heart rate, systolic blood pressure, diastolic blood pressure, mean blood pressure, oxygen saturation on pulse oximetry (SpO2), right atrium pressure, central venous pressure, temperature, oxygen saturation in arterial blood gas (SaO2), oxygen saturation in venous blood gas (SvO2)] in the full dataset captured from the bedside monitor and laboratory values. It then sends the data to the T3 platform to compute the IDO2 index in real time. IDO2 calculation is based on a model-based risk assessment methodology described and validated previously (9). The index is calculated at 5 second intervals and provides a continuous probability between 0 and 100 of the measured mixed systemic venous saturation (SvO2) being lower than 40% in the preceding time (30 min in our study). Continuous venous oximetry has been shown to improve outcomes in the Cardiac intensive care unit (CICU) and is recommended to be used to monitor patients' trajectories (10–12). It also provided information on oxygen transport dynamics, oxygen extraction ratio (OER), cardiac output, and oxygen delivery. It has been shown that a SVO2 of <40% or an OER of >50–60% have been associated with shock, lactic acidosis, and other worse outcomes (11–15). Hence IDO2 provides continuous estimation of patients' risks of experiencing inadequate oxygen delivery with intermittent measurement of some of the data variables like blood gases and SVO2. The IDO2 index can continue to be computed based on previously acquired data even when data is missing at particular points in time. This approach uses non-linear stochastic dynamic models of human physiology and recursive Bayesian estimation (9) (see Appendix for details on the model in Supplementary Materials). IDO2 has been previously validated demonstrating a positive correlation with other indices of oxygen perfusion (serum lactate) and adverse events (8, 9, 16).

The performance of this risk analytic algorithm hasn't been tested in patients presenting in a low resource setting. We hypothesize that despite the different disease spectrum and physiological states, the principles behind the physiological states are same, so we expect IDO2 index will perform same way in a phenotypically different population. This may stem from differences in disease type, more prevalence of malnutrition, and delayed presentation (condition that can alter the physiological state), thus making our cohort more unique than the one on which IDO2 was first validated.

## Methods

Retrospective review of prospectively collected medical records and T3 records of all patients (aged 1 day to 12 years) who received care in the CICU between October 1st, 2019, and October 1st, 2020, was done after approval approval by the ethical review committee of the Aga Khan University (application #2020-4795-10506). Our hospital is a tertiary care center in city of Karachi catering to a population of 47 million (Sind) and 12 million (Baluchistan) with multi-disciplinary/specialty adult and pediatric hospital. Annually ~250 open heart surgeries (including single and bi-ventricular repair) are done at our hospital. The CICU is a dedicated 4-bedded unit with 1:1 nurse-patient ratio. Average experience of bedside nursing care is 3 years with an attrition rate of 25%. The ICU is staffed by year 2 or 3 general pediatric post graduate trainees and four intensivists (one of them on call on a weekly basis). Our center is also part of international quality improvement collaborative (IQIC), and clinical data variables were extracted from this data. IQIC is a surgical database/registry that collects data from congenital heart disease surgical program in LMICs, for risk adjusted benchmarking of outcomes and quality improvement efforts.

Patients with SvO2 measurements with no associated IDO2 index in the preceding 30 min (minimum data set criteria for IDO2 index computation were not met), birth weight <2 kg, and who were born premature were excluded.

The T3 Data Aggregation and Visualization (T3) software is an FDA 510(k) approved application that collects, stores, and displays ICU data in near-real time. It employs proprietary data aggregation technologies developed by Etiometry (Etiometry Inc., Boston, MA) to enable the collection, segmentation, and patient binding of data to support care. T3 employs a web-based user interface to display data collected from monitoring devices as well as Etiometry algorithms anywhere within clinical workflows *via* a web browser. Data from patients' bedside monitor is continuously transferred to T3 platform every 5 s and it is stored in institutions' data warehouse which can be retrieved by authorized personnel.

T3 at Aga Khan University's CICU was implemented in November 2018. Initially only patient's bedside monitors and laboratory feed were connected and later data from ventilators were also captured. All stakeholders including physicians and nurses were trained on how to access and use T3. Nursing staff were also trained on appropriate labeling of blood samples for accurate feeding into the IDO2 algorithm. Regular spot checks and audits were done, which were verified by Etiometry Inc. as well. Once this routine practice was established, IDO2 calculation with full data set was started. During this period access to the platform for visualizing patient's data was provided to nurse leads, trainee, and attending physicians (intensivists, cardiologists, and surgeons). In October 2019 we installed persistent displays of T3 in our unit, so everyone had access to this platform and data. However, there was no compulsion to use this data and thus the information around use of this platform for clinical decision making wasn't measured for the purpose of this study.

All SvO2 measurements and preceding IDO2 data were extracted from the Etiometry's platform. Each SvO2 value was taken as an independent assessment of a patient's state of oxygen delivery at the time the measurement sample was collected. SvO2 measurements were done based on patients' clinical conditions and at the discretion of caring team. There was no set protocol used to decide when to obtain these labs. However, a general rule in our unit is that when patients are at risk of low cardiac output syndrome (LCOS) (i.e., prolonged cardiopulmonary bypass pump time) and arrhythmias, or have developed LCOS, SvO2 is measured. The average IDO2 Index was computed in the 30-min period immediately prior to each SvO2 measurement and used as a predictor score for SvO2 < 40%. The resulting receiver operating characteristic (ROC) curve was generated and the Area Under the Curve (AUC) was computed to demonstrate the overall discriminatory power of the index. AUC confidence intervals were generated using Bootstrapping, in which bootstrapped AUCs were calculated using random subsets of the data points selected with replacement from the original set. To test that increases in IDO2 values are associated with increased risk for SvO2 < 40%, the 30-min IDO2 values and its corresponding SvO2 measurements were divided into four bins based on average IDO2 value. The bins, which were chosen were chosen to divide the range of possible IDO2 values [0, 100] into four equal ranges, were 0–25, 25–50, 50–75, and 75–100. The absolute and relative risks for SvO2 < 40% were calculated for each bin. Each relative risk of SvO2 < 40% was calculated by computing the absolute risk of encountering SvO2 < 40% in a particular IDO2 bin relative to the absolute risk in the whole population. Finally, the increase in risk between adjacent bins were calculated and checked for statistical significance using a parametric method. Specifically, to test statistical significance of the increase, a *p*-value was calculated using a normal approximation for the distribution of the algorithm of the ratio of risk in adjacent bins. Results are presented as median with interquartile range (IQR) for continuous data and frequencies with percentages for categorical variables. Relative risk along with *p*-value for SvO2 < 40% for different increasing values of IDO2 in the preceding 30-min average IDO2 value are reported.

## Results

A total of 69 CICU admissions were identified from 65 patients. Median age of the study population was 9.3 (IQR 4–24.7) months. Sixty-four (92.8%) admissions had a cardiac diagnosis; 61/64 (95.3%) were admitted after cardiac surgery. Admitting diagnosis included tetralogy of Fallot (23, 33.3%), ventricular septal defect (17, 24.6%), transposition of great arteries (5, 7.2%), coarctation of Aorta (4, 5.8%) and others (20, 27.5%). The median stay was 6 days in the CICU (IQR 1–6) and 9 days in the hospital (IQR 1–9). Mortality was 10.1%. There were a total of nine CPR events amongst the included patients. Of the seven patients who expired; three had CPR preformed on them while the remaining four were in comfort care with a do not resuscitate directive (Table 1).

**Table 1:** Demographic details of study population (*n* = 69).

**Demographics**			**Frequency (%)**	**Median (IQR)**
Gender	Male		46 (66.7)	
	Female		23 (33.3)	
Age (month)				9.3 (20.8)
Admission Type	Medical		8 (11.6)	
	Surgical		61 (88.4)	
Diagnosis	Cardiac		64 (92.8)	
	1. Tetralogy of fallot		1. 23 (33.3)	
	2. Ventricular septal defect		2. 17 (24.6)	
	3. Transposition of great arteries		3. 5 (7.24)	
	4. Coarctation of aorta		4. 4 (5.80)	
	5. Atrial septal defect		5. 1 (1.45)	
	6. Others		6. 14 (20.3)	
	Non-cardiac		5 (7.2)	
Outcome	Survived		62 (89.9)	
	Expired		7 (10.1)	
Length of mechanical ventilation (hours)				43.5 (79)
Length of CICU stay (days)				6 (6)
Length of hospital stay (days)				9 (9)
CPR	Yes		9 (13.0)	
	No		60 (87.0)	
Duration of CPR (*n* = 9) (minutes)				7 (11)
Scoring of cardiac procedures **(*****n*** **=** **60)**	RACHS	1	16 (26.7)	
		2	31 (51.7)	
		3	8 (13.3)	
		4	5 (8.33)	
	STAT	1	21 (30.4)	
		2	22 (36.7)	
		3	7 (11.7)	
		4	10 (16.7)	

*IQR, interquartile range; CICU, cardiac intensive care unit; CPR, cardiopulmonary resuscitation; RACHS, risk adjustment in congenital heart surgery; STAT, The Society of Thoracic Surgeons-European Association for Cardio-Thoracic Surgery*.

**Figure 1:**
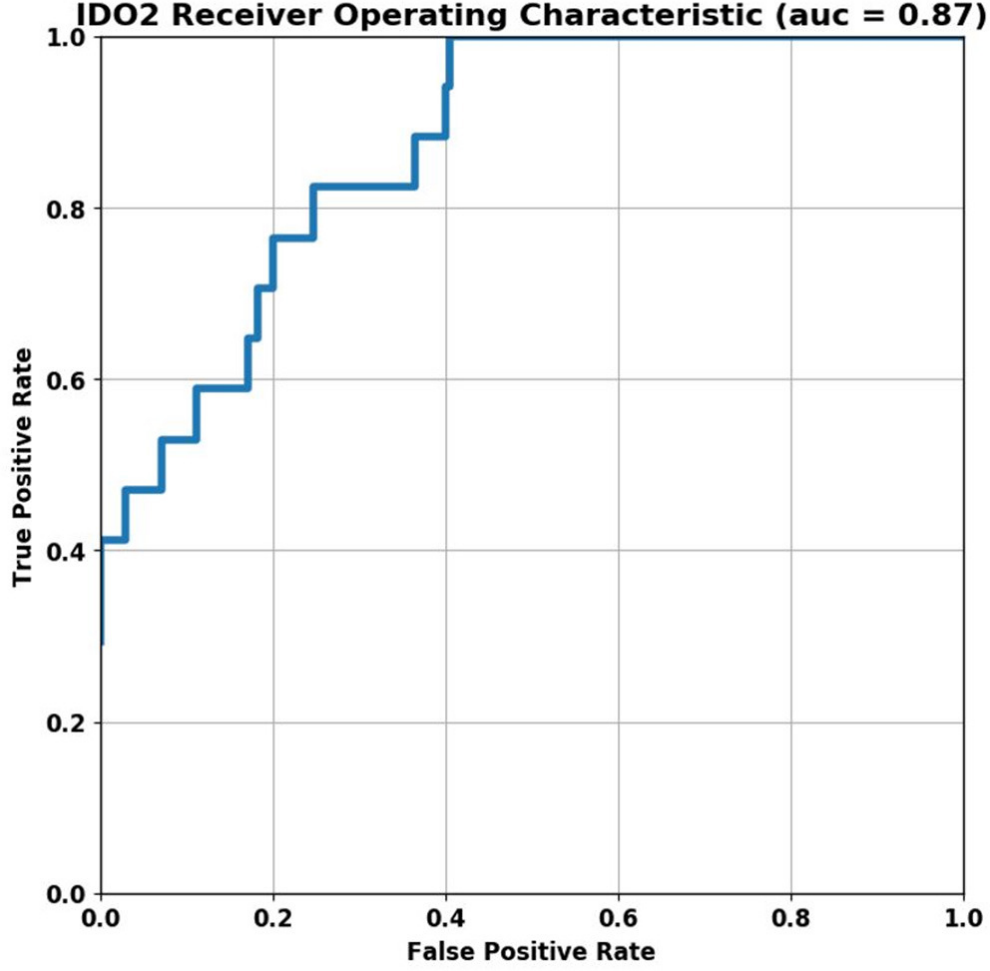
ROC curve of detecting SvO2 < 40% using 30-min IDO2 average.

A total of 194 SvO2 labs were taken during the study period. Seven (3.6%) SvO2 labs were excluded for insufficient data required for IDO2 calculation. Of the 187 (96.4%) total included SvO2 labs, 17 (9%) were <40%. Sixty-three patients had IDO2 values in the bin of <25, 11 patients had values between 25 and 50, 10 between 50 and 75, 9 had IDO2 >75. The AUC of predicting SvO2 < 40% using 30-min average IDO2 was 0.87 (confidence interval: 0.79–0.94) (Figure 1). Average IDO2 ≤ 25 had the lowest risk of SvO2 < 40% (relative risk: 0.33, confidence interval: 0.11–0.96, *p*-value 0.042) relative to all other average IDO2 ranges. There was no significant difference in the relative risk of SvO2 < 40% when comparing IDO2 ≤ 25 with 25 < IDO2 ≤ 50 and 25 < IDO2 ≤ 50 with 50 < IDO2 ≤ 75 (Table 2).

**Table 2:** Absolute risk and relative risk of experiencing SvO2 < 40% for each IDO2 bin.

**Testing parameter**	**Averaging period (minutes)**	**Number of patients**	**IDO2 range**	**Absolute risk**	**# SvO2**	**# SvO2 < 40%**	**Relative risk**	***p*-value**
IDO2	30	69	All	9.09 (5.39–14.16)	187	17		
IDO2	30	63	0_to_25	3.01 (0.83–7.52)	133	4	0.33 (0.11–0.96)	0.042
IDO2	30	11	25_to_50	14.29 (1.78–42.81)	14	2	1.57 (0.40–6.13)	0.515
IDO2	30	11	50_to_75	14.29 (3.05–36.34)	21	3	1.57 (0.50–4.92)	0.4377
IDO2	30	9	75_to_100	42.11 (20.25–66.50)	19	8	4.63 (2.31–9.28)	<0.0001

**Figure 2:**
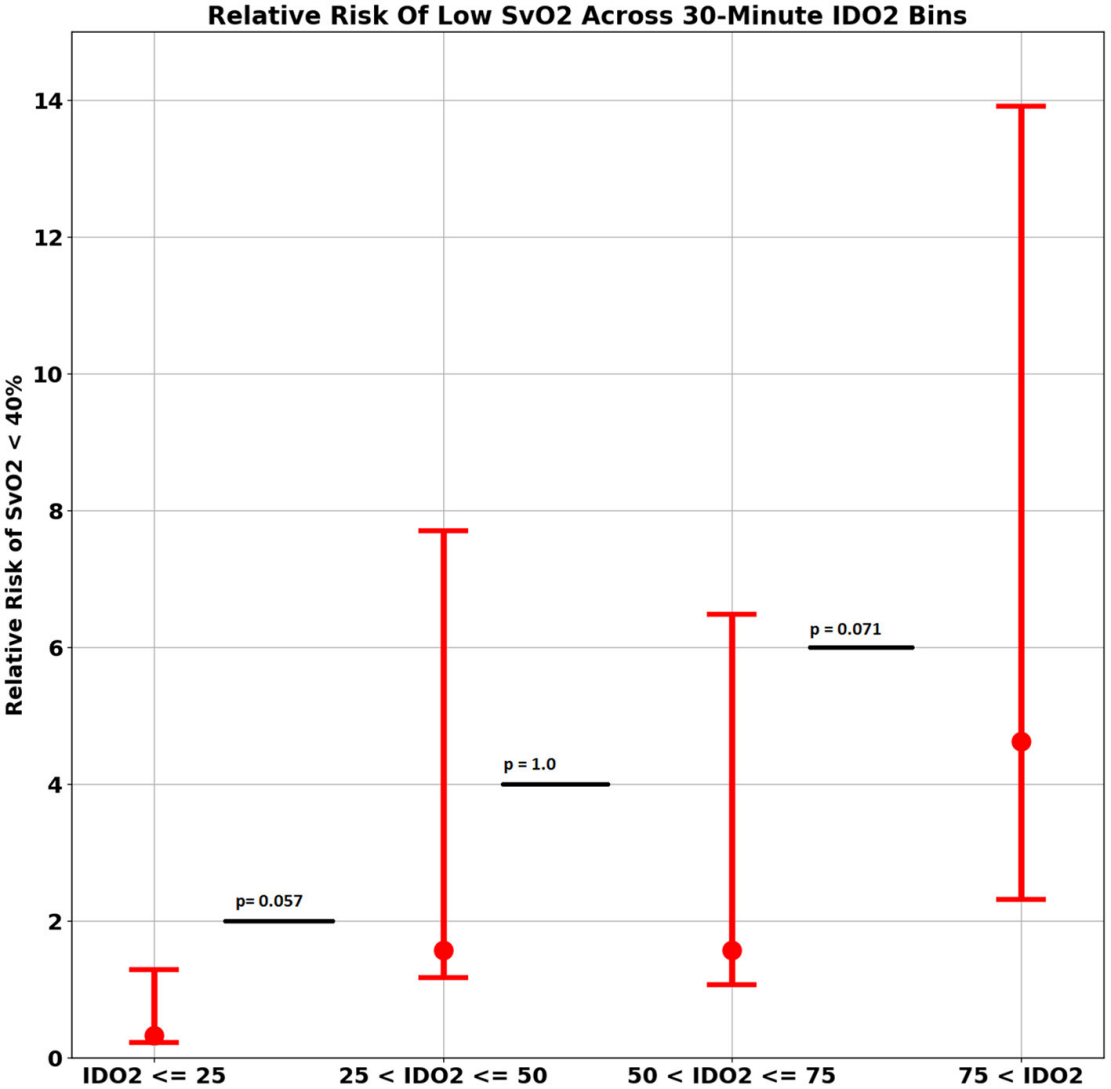
Relative risk of low SvO2 across 30-min IDO2 Bin.

Compared to all other IDO2 ranges, average IDO2 above 75 had the highest absolute risk (42.11, confidence interval: 20.25–66.50) and the highest relative risk (4.63, confidence interval: 2.31–9.28, *p*-value < 0.0001) of SvO2 < 40% (Figure 2).

## Discussion

In pediatric post-operative CHD patients from a CICU in a low resource setting, IDO2 (an FDA approved algorithm) demonstrated modest ability to estimate the risk for low SvO2 (a surrogate of low cardiac output and tissue perfusion). The AUC value of 0.87 for predicting SvO2 < 40% in our cohort is better than the previously published initial results where the AUC was 0.79 (CI 0.76–0.82). This could be due to the inherent property of iterative algorithms which improves as more data is provided and analyzed. The previous work of assessing the performance of IDO2 has been performed in centers present in high income regions (HIR) (8, 9, 17). To the best of our knowledge this is probably the first study demonstrating performance of this algorithm on post-operative patients in LMICs. CHD patients in LMICs are considered to have a very different spectrum of disease (2, 5, 6, 18, 19). These patients present late and majority of the time have associated co-morbidities like infections, severe malnourishment, and significant lung injury (especially in shunt lesions) due to repeated pneumonia (20–25). These patients thus have different physiological states than similar patients presenting in HIR (i.e., advanced pulmonary vascular obstructive disease, significantly elevated filling pressures due to prolonged volume load etc.) (26). IDO2 also has the ability to use minimum data set (heart rate, SpO2 and blood pressure) to continuously estimate the risk for SvO2 < 40%, all of which can be taken non-invasively. This is also very contextual for LMICs where cost of care is a huge issue. It is thus encouraging to see that the performance of IDO2 is modest even in a very different patient population thus adding to the geographical validation of this tool.

The ability of IDO2 in predicting adverse events like cardiac arrest has been demonstrated by Futterman et al. The IDO2 measured in the preceding 120 min of the event had an AUC of 0.74 which improved to 0.81 when single ventricle patients were included in predicting a cardiac arrest in these patients (17). We were unable to perform such analyses as there were only nine CPR events, and we only had a small proportion of single ventricle patients in our cohort.

Our results also depict that IDO2 < 25 had the lowest risk of having SvO2 < 40%, while this risk increases with increasing IDO2 values and reaches maximum with IDO2 > 75. These findings are similar to what has been shown by Futterman et al. (17). This can also be used to make a framework to trigger clinical teams for any intervention. So far we don't know which threshold of IDO2 value to get an alert or to act on, as it is very tough to decide at what IDO2 a particular intervention should be aimed at. Another study by Rogers et al.; who compared the IDO2 and LCOS score to predict adverse outcomes in their post CHD surgery patients; showed that LCOS score had a stronger association with specified medium term adverse outcomes compared to IDO2 (8). This might be due to very low frequency of adverse events in their population (7%). Further work will be needed to test the ability of IDO2 vs. other conventional measures of adverse event prediction (LCOS score, vasoactive inotropic score, pediatric risk of mortality etc.) especially in settings where such events have occurrences as high as we had in our population (i.e., 10% mortality rate).

Nearly 90% of all CHD patients are present in what is considered a low resource setting, especially LMICs (19). Each of these countries face similar challenges of lower expertise among staff which is compounded with additional complexities in patients (delayed presentation and or diagnosis, lack of awareness among primary physicians, less availability of treating facilities) and financial constraints; when compared to HIRs (5). As such, automated tools to help identify risks in critically ill patients may provide health care workers in LMICs with a decision support system, thus helping them deliver higher quality care. Since the mortality and frequency of adverse events in such settings is higher; such tools may have a better performance in helping improve the care and translate in better patient outcomes. To make them useful in such settings, a clear framework of implementation, standard operating procedures, and above all, compliance to these management protocols centered around such risk analytic tools is critical and may be the missing piece in demonstrating the true benefit of such tools in improving patient outcomes. Previously such a beneficial effect on patient outcome has been demonstrated after implementation of the T3 platform in a high resource setting (27).

There are several limitations of our study. Firstly, it is a single center study with a small sample size and thus precluded more detailed analysis and demonstration of significance in some of the results. Since the number of patients in this study were low, we took each IDO2 value as an independent value for IDO2 estimation. Another limitation is that its effects on patient care processes, the ultimate goal of such algorithms, was not studied. Though majority of the cohort were post-operative CHD patients, there were some non-cardiac critically ill patients admitted to CICU who were also included. However, the results are still a giant leap in application of this algorithm beyond neonatal population and in a different geographic location with different characteristics. We understand that this is not a true validation study, rather a pilot study, but we strongly feel that once incorporated into the normal work-flow of patient care delivery, such a model based algorithm and data visualization platform can transform the care processes and outcomes in resource limited settings.

## Conclusions

A physiologic model-based risk analytic algorithm performed well in a different population subset after successful implementation in low resource setting.

## Data Availability Statement

The original contributions presented in the study are included in the article/Supplementary Material, further inquiries can be directed to the corresponding author.

## Ethics Statement

The studies involving human participants were reviewed and approved by Ethical Review Committee of the Aga Khan University (Ref # 2020-4795-10506). Written informed consent from the participants' legal guardian/next of kin was not required to participate in this study in accordance with the national legislation and the institutional requirements.

## Author Contributions

QA and BH conceived the idea and lead the implementation. QA trained all staff and did analysis. NS helped in sustaining the implementation. MH and FS collected data along with QA. All authors contributed to the article and approved the submitted version.

## Conflict of Interest

The authors declare that the research was conducted in the absence of any commercial or financial relationships that could be construed as a potential conflict of interest.

## Publisher's Note

All claims expressed in this article are solely those of the authors and do not necessarily represent those of their affiliated organizations, or those of the publisher, the editors and the reviewers. Any product that may be evaluated in this article, or claim that may be made by its manufacturer, is not guaranteed or endorsed by the publisher.
