# Supplementary material for: Performance of a Risk Analytic Tool (Index of Tissue Oxygen Delivery “IDO2”) in Pediatric Cardiac Intensive Care Unit of a Developing Country
Source: Front Pediatr. 2022 Jun 3;10:846074. doi: 10.3389/fped.2022.846074 (PMC9203960; doi:10.3389/fped.2022.846074)
Supplement: Supplementary file 1 [file Data_Sheet_1.PDF]

Appendix:

A. IDO2 data source and calculation details:

IDO2 provides probability of a patient experiencing inadequate oxygen delivery, which is defined as mixed venous oxygen saturation (SvO2) <40%. SvO2 is used as a surrogate for cardiac output and oxygen delivery and provides action able information regarding patients' physiology.(1-4) It has shown to be associated with adverse outcomes in children after congenital heart surgery.(1-3) The IDO2 index has a range between 0 to 100, increasing value means there is an increasing risk of inadequate oxygen delivery. Since it is being computed continuously, it provides timely insight into patients' physiology requiring attention.

IDO2 index is computed based on 10 physiologic measures and laboratory tests results (which is collected in an automated way by the T3 software). (Table 1) This is full data set.

Table 1: List of Measures used to compute IDO2

**Variable**	**Measurement Source**
Heart Rate	HR (ECG)
Systolic Blood Pressure	(ABPs/ARTs) Arterial line
	(NiBPs) Blood Pressure Cuff
Mean Blood Pressure	(ABPs/ARTs) Arterial line
	(NiBPs) Blood Pressure Cuff
Diastolic Blood pressure	(ABPs/ARTs) Arterial line
	(NiBPs) Blood Pressure Cuff
Filling Pressure	CVPm (Central venous pressure)
	RAPm8 (Right atrium pressure)
SpO2	Pulse Oximeter
Hemoglobin	Laboratory
SvO2	Venous blood gas
SaO2	Arterial blood gas
Temperature	Temperature Probe (Skin, rectal)

However, IDO2 can be computed with a minimum data set (minimum requirement of IDO2 computation).

a. Heart rate from ECG or pulse at a minimum of once every 60 seconds
b. SpO2 from pulse oximetry at a minimum of once every 10 minutes
c. Blood Pressure (mean/diastolic/systolic) at a minimum of once every 10 minutes

This means it can be calculated even without SvO2.
T3 software collects patients physiologic and laboratory data continuously, the IDO2 algorithm uses Bayes theorem to interpret the newly acquired data to give its own assessment of the physiologic state of the patient. It then uses the interpreted data to update its assessment of the patient physiologic state. The patient state is represented as a set of patient physiologic variables, each of which is modeled with a probability distribution to account for patient-to-patient variations and measurement uncertainty. Physiologic variables representing the patient state are also related to one another using

established relationship of human physiology.(5) It also incorporates models of the measurement in collection devices and sensors that account for the potential errors in the data. Some of the parameters used in model are continuously dependent on the patient age. T3 data computes the IDO2 index by calculating the cumulative probability of the SvO2 probability distribution between 0% and 40%. As a result of the model-based approach by which IDO2 algorithm is computed, the algorithm exhibits following properties:

A. The IDO2 index can continue to be computed based on previously acquired data, even when data is missing at particular points in time.
B. The algorithm can incorporate and reconcile multiple measurements of the same physiologic variables (for example heart rate derived from ECG and arterial line waveform) since it accounts for the noise and common errors inherent in each possible measurement source
C. The algorithm analyzes the likelihood of a particular measurement values given other collected data values and its current patients state assessment and can reject potential measurement artifacts that can contradict the physiology model or the other physiologic measurements (for example, interruption of measurement to flush the line or to obtain laboratory tests).

The model incorporates following effects:

1. The nominal value of oxygen consumption used by the model depends on the current body temperature
2. Heart rate, systemic vascular resistance and unstressed blood volume are affected by the autonomic regulation through a model of the baroreflex mechanism
3. Pulse pressure depends on stroke volume, systolic duration and systemic vascular resistance
4. Stroke volume depends on mean arterial pressure, central venous pressure, ventricular function, and systolic duration
5. The model accounts for a potential persistent bias between arterial oxygen saturation derived from arterial blood gas and pulse oximetry.

B. Data collection for this study:

1. Physiologic monitor data were streamed directly into the T3 database. Physiologic data (vital signs like heart rate, blood pressure etc) were retrieved from the T3 software (Etiometry platform). When a new laboratory message is received from the laboratory system, it is time tagged, displayed, and stored relative to the continuous vital signs.
2. IDO2 was computed for each patient, 30 minutes before the next SvO2 lab test. The laboratory data were collected, and time aligned with the physiologic data automatically by the T3 software.
3. Clinical data was retrieved from the IQIC data base kept in the institution. This data was collected prospectively from patients' medical record and entered in IQIC database.
4. All measurements are not collected at every point during the patients' stay. Inherent to the recursive Bayesian algorithm that is behind the iDO2 calculation (i-e the Kalman filter), it has the ability to still provide an estimate of the underlying patient state, in the absence of a particular measurement at a given time slice. This is achieved through a dynamics model which specifies how the patient state evolve in time, as well as how the associated uncertainty evolves. The IDO2 algorithm naturally handles missing data of this type and will calculate and report a value

for varying levels of monitoring as long as minimum set of data is available which is mentioned above.

IDO2 Validation:

IDO2 was validated in a retrospective study of children aged 0 days till 12 years, with a sample size of 2282 patients. For design and development of this IDO2 algorithm, data from 780 patients was used and it was then validated on 1502 patients from 2011 to 2016. The study used periodic measurements of mixed venous oxygen saturation as proxies of the actual mixed venous oxygen saturation. For each SvO2proxy measurement in the first 10 postoperative days, the average IDO2 Index was computed 30 minutes immediately prior to the measurement and used as a predictor score for SvO2proxy $< 40\%$. The resulting Receiver Operating Characteristic (ROC) curve was generated and the Area Under the Curve (AUC) was computed. SvO2proxy less than 40% was chosen as a comparative metric because mixed venous oxygen saturation levels below this have been documented to correspond with inadequate oxygen delivery levels. (1, 2, 4, 6)

In results, three data sets were examined. In the first dataset, full measurement data composed of all possible measures listed above were used by the algorithm for the IDO2 computation, which included past mixed venous oxygen saturation measurements. In the second dataset, a "medium" dataset was curated by removing all the SvO2 lab measurements. In the third dataset, only minimum measurement dataset was used by the algorithm for the computation which included heart rate every 60 seconds, SpO2 every 10 minutes and blood pressure every 10 minutes. The area under the curve (AUC) for the prediction with the full set of data with 30-minute averaging is AUC = 0.83 with 95% confident interval of 0.82-0.85. The AUC for using the medium set of data with 30-minute averaging is AUC = 0.78 with 95% confident interval of 0.76-0.80. The AUC for the minimum set of data with 30-minute averaging is AUC = 0.76 with a 95% confidence interval of 0.75- 0.78.

References:

1. Hoffman GM, Ghanayem NS, Kampine JM, Berger S, Mussatto KA, Litwin SB, et al. Venous saturation and the anaerobic threshold in neonates after the Norwood procedure for hypoplastic left heart syndrome. Ann Thorac Surg. 2000;70(5):1515-20; discussion 21.
2. Hoffman GM, Mussatto KA, Brosig CL, Ghanayem NS, Musa N, Fedderly RT, et al. Systemic venous oxygen saturation after the Norwood procedure and childhood neurodevelopmental outcome. J Thorac Cardiovasc Surg. 2005;130(4):1094-100.
3. Bronicki RA. Venous oximetry and the assessment of oxygen transport balance. Pediatr Crit Care Med. 2011;12(4 Suppl):S21-6.
4. KR W. Use of central venous oxygen saturation to guide therapy. Am J Respir Crit Care Med. 2011;184(5):514-20.
5. Baronov D, McManus M, Butler E, Chung D, Almodovar MC. Next generation patient monitor powered by in-silico physiology. Annu Int Conf IEEE Eng Med Biol Soc. 2015;2015:4447-53.
6. Martin J, Shekerdemian LS. The monitoring of venous saturations of oxygen in children with congenitally malformed hearts. Cardiol Young. 2009;19(1):34-9.
